# Case Report: Splenic Irradiation for the Treatment of Chronic Active Antibody-Mediated Rejection in Kidney Allograft Recipients With *De Novo* Donor-Specific Antibodies

**DOI:** 10.3389/fimmu.2021.661614

**Published:** 2021-04-15

**Authors:** Lan Zhu, Zhiliang Guo, Rula Sa, Hui Guo, Junhua Li, Gang Chen

**Affiliations:** ^1^ Tongji Hospital, Tongji Medical College, Institute of Organ Transplantation, Huazhong University of Science and Technology, Wuhan, China; ^2^ Key Laboratory of Organ Transplantation, Ministry of Education and Ministry of Public Health, Chinese Academy of Medical Sciences, Wuhan, China; ^3^ Department of Nephrology, Tongji Hospital, Tongji Medical College, Huazhong University of Science and Technology, Wuhan, China

**Keywords:** chronic active antibody-mediated rejection (cABMR), *de novo* donor-specific antibody, splenic irradiation, renal transplantation, case report

## Abstract

Chronic active antibody-mediated rejection (AMR) in renal transplantation is usually refractory to current conventional treatment with rituximab, plasmapheresis (PP), and intravenous immunoglobulins (IVIG). Splenic irradiation has been reported to be effective in the rescue of early severe acute AMR after kidney transplantation; however, its effect in chronic active AMR has not been reported to date. In order to reduce donor-specific antibody (DSA) and prevent the progression of chronic AMR, we used repetitive low-dose splenic irradiation, together with rituximab and PP/IVIG, in two living-related kidney transplant recipients with pathologically diagnosed chronic active AMR and the presence of long-term class II-*de novo* DSA. DSA monitoring and repeated renal biopsy revealed significantly reduced DSA levels as well as alleviated glomerulitis and peritubular capillaritis in both patients after treatment, and these therapies may have played a role in delaying the progression of chronic AMR. Although DSA levels in both patients eventually rebounded to some extent after treatment, serum creatinine increased slowly in one patient during the 16-month follow-up period and remained stable in the other during the 12-month follow-up period. Given the poor efficacy of conventional treatment at present, splenic irradiation may still be one of the treatment options for chronic active AMR.

## Introduction

Chronic active antibody-mediated rejection (AMR) has long been recognized as the leading cause of late allograft loss in kidney transplantation (1, 2). With the widespread adoption of single-antigen beads testing and the use of electron microscopic analysis, more and more cases of chronic active AMR have been clearly and precisely diagnosed. However, the treatment of chronic active AMR is still an unmet medical need. Current standard therapy, including rituximab plus plasmapheresis (PP)/intravenous immunoglobulins (IVIG), has generally given unsatisfactory results (3, 4). Even the most promising anti-IL-6 receptor antibodies have had controversial effects, according to the latest publication (5).

Historically, splenectomy was routinely performed in ABO-incompatible kidney transplantation (6), and it has been further used with success in the rescue of early severe acute AMR (7–9). In a recently published case report, splenic irradiation was added to the conventional treatment (PP/IVIG, rituximab, and eculizumab) to rescue early severe acute AMR in two kidney transplant recipients, achieving excellent therapeutic effects in both patients (10). Owing to the difference in immunological mechanisms between late chronic AMR and early acute AMR, whether splenic irradiation can also play an important complementary role in the treatment of chronic active AMR remains to be determined. In the present study, we describe for the first time the clinical course and efficacy of spleen irradiation as an adjunctive therapy in two kidney transplant recipients with chronic active AMR.

## Donor-Specific Antibody (DSA) Monitoring

Sera were screened for HLA antibody using HLA class I and II single-antigen beads (LABScreen™ Single Antigen Beads, One Lambda Inc., Canoga Park, CA). In brief, 20 µl of 1:3 diluted sera were added to 2.5 µl of antigen beads, incubated in the dark for 30 min at room temperature, and then washed with a wash buffer. One hundred microliters of PE-conjugated goat anti-human IgG second antibody was added to the beads and the mixture was incubated for 30 min in the dark at room temperature, washed, and read on a LABScreen™100 Luminex. Antibodies were detected by measuring the mean fluorescence intensity (MFI) of each single-antigen bead. Adjusted raw MFI values >1000 were defined as positive reactions.

In addition, in order to avoid antigen saturation, we tested the serum samples at 1:6 dilution when the MFI value from the 1:3 diluted serum was >15,000. The samples were diluted with phosphate-buffered saline.

## Complement-Dependent Cytotoxicity (CDC) Assays Using Flow Cytometry

CDC assays were assessed by flow cytometry using donor lymphocytes isolated from peripheral blood as the target cells, as described previously (11, 12). The cells were analyzed using a FACSCalibur with Cellquest Pro 6.0 software (BD Biosciences, USA). The percentage of propidium iodide-positive cells was used to determine the extent of the CDC. Cells incubated with neither human serum nor rabbit complement (cells only) and cells incubated with rabbit complement alone (cells + complement) served as negative controls.

## Case 1

**Figure 1:**
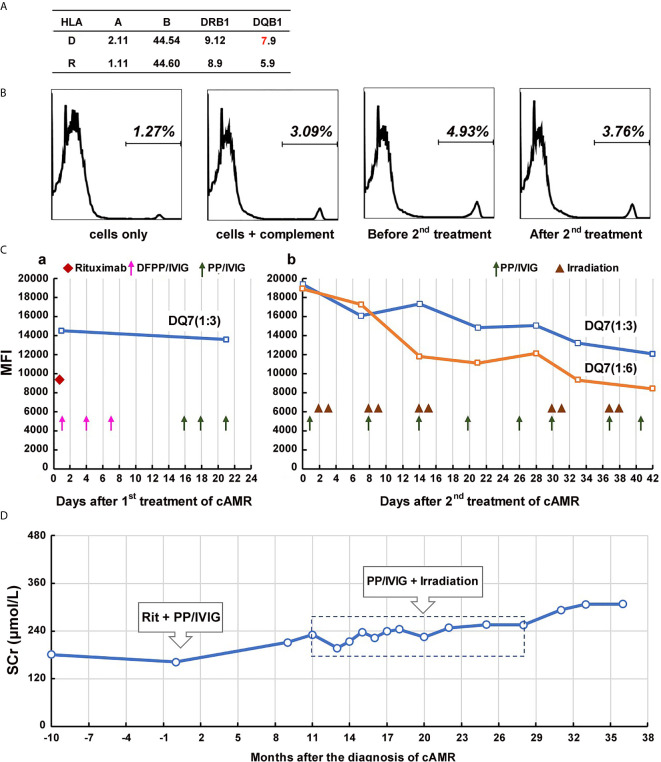
Course of Patient 1’s DSA and serum creatinine after the diagnosis of chronic active AMR. (A) HLA phenotype results of the patient 1 and his donor. **(B)** Flow-CDC results before and after the second treatment of chronic AMR. Donor lymphocytes incubated with neither the patient’s serum nor rabbit complement (cells only) and cells incubated with rabbit complement alone (cells+complement) served as negative controls. **(C)** changes in MFI values of donor-specific anti-DQ7 antibody (1:3 or 1:6 diluted serum) during the first round of treatment **(a)** and the second round of treatment **(b)**. **(D)** Changes of serum creatinine levels before and after the diagnosis/treatment of chronic active AMR.

The patient is a 38-year-old man who underwent living-related kidney transplantation for his IgA nephropathy 8 years ago (January 2013) following 4 years of hemodialysis. The donor was his father (59 years old at that time). The donor-recipient HLA-A, -B, -DR, -DQ mismatch grade was 4 (**Figure 1A**). The pre-transplant panel reactive antibodies (PRA) and complement-dependent cytotoxicity (CDC) test results were both negative. Given the low immunological risk, the patient did not receive any induction therapy. He recovered smoothly after the operation, and the renal allograft function soon returned to normal. Since the transplant, this patient has received triple maintenance immunosuppressive therapy with oral tacrolimus, mycophenolate mofetil, and prednisone.

**Figure 2:**
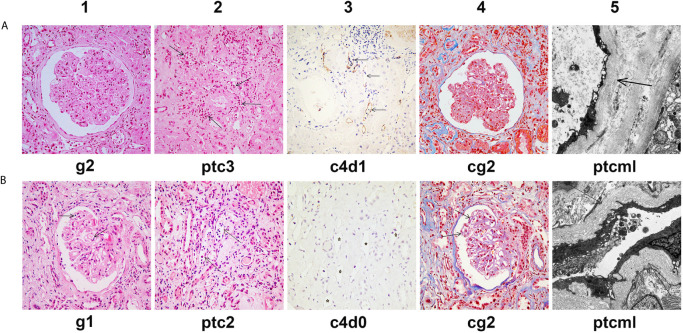
Histological features of chronic active AMR in Patient 1 before and after treatment. **(A)** Typical histological findings of chronic AMR in Patient 1 before the first round of treatment and their Banff 2017 Scores. **(B)** Histological findings of renal allograft in Patient 1 at the 14th month after the second round of treatment and their Banff 2017 Scores. Panel A1 and B1 (hematoxylin and eosin, 400x magnification) show glomerulitis. Panel A2 and B2 (hematoxylin and eosin, 400x magnification) show peritubular capillaritis (arrow). Panel A3 and B3 (immunohistochemical staining, 400x magnification) show peritubular capillary C4d deposition (arrow). Panel A4 and B4 (Masson’s trichrome, 400x magnification) show transplant glomerulopathy, characterized by focal glomerular basement membrane double contours. Panel A5 (electron microscopy, 2000x magnification) shows 5-7 circumferential layers of peritubular capillary basement membrane multilayering (ptcml, arrow). Panel B5 (electron microscopy, 3000x magnification) shows 8-10 circumferential layers of peritubular capillary basement membrane multilayering (ptcml, arrow).

Beginning 2 years after the transplantation, the patient’s serum creatinine level started to increase slowly, and proteinuria began to appear. Five years after transplantation, the patient underwent renal biopsy because of significantly elevated serum creatinine (181 μmol/L) and severe proteinuria (2,260 mg/24h). The pathology results showed moderate glomerulitis (g2), severe peritubular capillaritis (ptc3), mild peritubular capillary C4d deposition (C4d1), focal glomerular basement membrane double contours (cg2), peritubular capillary basement membrane multilayering (electron microscopy), and positive IgA deposition (++), which were diagnosed as mild chronic active AMR (Banff 2017 Schema), transplant glomerulopathy (TG), and recurrent IgA nephropathy (**Figure 2A**). Meanwhile, the single-antigen bead assay detected anti-DQ7 DSA at an MFI value of 14,506. Upon admission, the patient received his first round of treatment for chronic AMR: rituximab (200 mg), three sessions of double-filtration plasmapheresis (DFPP)/IVIG (25g each time), followed by three sessions of PP/IVIG (25g each time). After the treatment, DSA detection showed no obvious decease in anti-DQ7 antibody MFI (13,576) (**Figure 1C-a**). Eight months later, the anti-IL-6 receptor monoclonal antibody (tocilizumab, 400 mg) was begun and scheduled to be given monthly. However, the treatment was terminated one month later because of the development of severe leukopenia (white cell count: 2.8 x 10^9^/L). After that, we did not give the patient specific treatment of AMR.

At 19 months after the first round of treatment (6 years and 7 months after transplantation), the patient’s serum creatinine continued to rise (225 μmol/L), there was aggravation of the proteinuria (3,225 mg/24h), and the MFI of the anti-DQ7 DSA further increased to 19,399. Flow-CDC testing using isolated fresh donor lymphocytes as target cells showed a weakly positive result (4.93%) as compared to the negative controls (cells only: 1.27%; cells + complement: 3.09%) (**Figure 1B**). Therefore, the patient was hospitalized again and received his second round of treatment: eight PP/IVIG sessions in combination with splenic irradiation (10 times, 50 cGy each time, 500 cGy in total) within a 42-day course (**Figure 1C-b**). He tolerated the treatment well, without significant gastrointestinal problems or any signs of myelosuppression, and showed only mild fatigue. After the treatment, the MFI of the anti-DQ7 DSA decreased from 19,399 to 12,061 (1:3 diluted serum). When we further diluted the sera samples to 1:6, the MFI value of the DSA demonstrated a more obvious reduction (from 18,914 to 8,405) (**Figure 1C-b**). Meanwhile, the Flow-CDC result decreased to 3.76%, which was very close to the negative control value.

The patient has further been followed up for 16 months since the second round of treatment. His serum creatinine remained relatively stable for the first 10 months (248-255 μmol/L) (**Figure 1D**). However, from the 11th month of follow-up, the serum creatinine gradually increased to >300 μmol/L (**Figure 1D**), the proteinuria increased (5,346 mg/24h), and the MFI of the DQ7-DSA rebounded to 14,923. At the 14th month of follow-up, the patient underwent another renal biopsy. Results showed that the acute tissue injury was alleviated (g1 and ptc2) as compared with earlier examinations, and the C4d deposition in peritubular capillaries had become negative, but the chronic tissue lesions were slightly worse (**Figure 2B**). In the latest follow-up, the patient showed mild edema, but his general condition was good. Biochemical examination of his blood revealed decreased levels of total protein (54 g/L) and albumin (29 g/L) and elevated levels of serum creatinine (316 μmol/L) and BUN (24.4 mmol/L).

## Case 2

**Figure 3:**
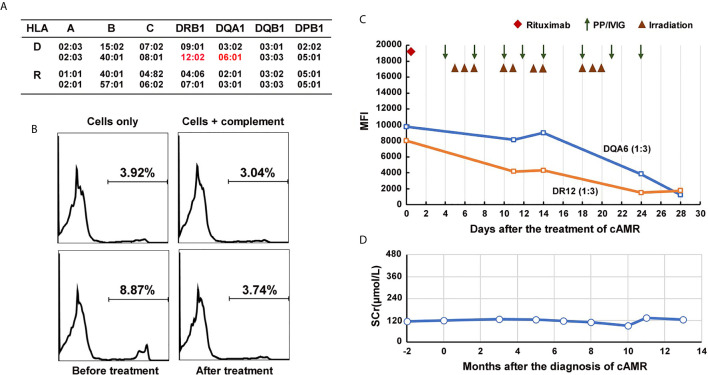
Course of Patient 2’s DSA and serum creatinine after the diagnosis of chronic active AMR. **(A)** HLA genotype results of the patient 2 and his donor. **(B)** Flow-CDC results before and after the treatment of chronic AMR. Donor lymphocytes incubated with neither the patient’s serum nor rabbit complement (cells only) and cells incubated with rabbit complement alone (cells +complement) served as negative controls. **(C)** changes in MFI values of donor-specific anti-DR12 and anti-DQA6 antibodies (1:3 diluted serum) during the treatment. **(D)** Changes of serum creatinine levels after the diagnosis and treatment of chronic active AMR.

The patient is a 44-year-old man who received a living kidney transplant from his 34-year-old wife in June, 2012 after 1.5 years of hemodialysis. The donor-recipient HLA-A, -B, -C, -DR, -DQA, -DQB, -DP mismatch grade of allele-level genotypes was 9 of 14 (**Figure 3A**). The pre-transplant PRA and CDC were both negative. His serum creatinine normalized after transplantation without any special events. Since the transplant, the patient has received a tacrolimus-based triple maintenance immunosuppressive therapy to prevent rejection.

**Figure 4:**
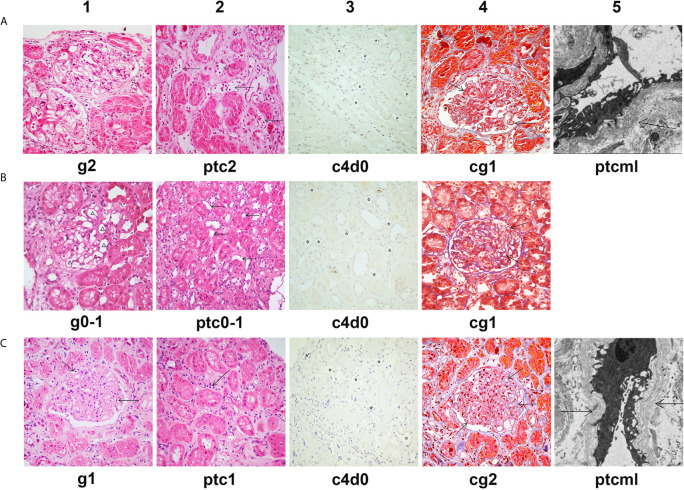
Histological features of chronic active AMR in Patient 2 before and after treatment. **(A)** Typical histological findings of chronic AMR in Patient 2 before the treatment and their Banff 2017 Scores. **(B)** The pathology results of a second renal biopsy performed after 4 weeks of treatment and their Banff 2017 Scores. **(C)** The pathology results of a third renal biopsy performed at the tenth month after the treatment and their Banff 2017 Scores. Panel A1, B1 and C1 (hematoxylin and eosin, 400x magnification) show different degrees of glomerulitis. Panel A2, B2 and C2 (hematoxylin and eosin, 400x magnification) show different degrees of peritubular capillaritis (arrow). Panel A3, B3 and C3 (immunohistochemical staining, 400x magnification) show no C4d deposition in peritubular capillaries (asterisk). Panel A4, B4 and C4 (Masson’s trichrome, 400x magnification) show different degrees of transplant glomerulopathy, characterized by focal glomerular basement membrane double contours (arrow). Panel A5 (electron microscopy, 2500x magnification) and Panel C5 (electron microscopy, 4000x magnification) show 5-7 circumferential layers of peritubular capillary basement membrane multilayering (ptcml, arrow).

In September 2019, the patient underwent a biopsy of his renal allograft because of proteinuria (676 mg/24h). The results showed moderate glomerulitis (g2) and peritubular capillaritis (ptc2), focal glomerular basement membrane double contours (cg1), peritubular capillary basement membrane multilayering (electron microscopy), IgA deposition (++), and focal glomerular C4d deposition, but no peritubular capillary C4d deposition (C4d0), all of which indicated mild chronic active AMR and recurrent IgA nephropathy (**Figure 4A**). Meanwhile, the single-antigen bead assay detected *de novo* DSA against DR12 (MFI: 8,023) and DQA6 (MFI: 9,780). The Flow-CDC test using isolated fresh donor lymphocytes as target cells showed a mildly positive result (8.87%) that was higher than that of the negative controls (cells only: 3.92%; cells + complement: 3.04%) (**Figure 3B**). To treat the chronic AMR, the patient was given rituximab (200 mg), eight PP/IVIG (20g each time) sessions (every 2-4 days), and splenic irradiation (10 times, 50 cGy each time, 500 cGy in total) (**Figure 3C**). After 4 weeks of treatment, the MFI levels of DSA significantly decreased (1:3 diluted serum; DR12-MFI: 1,742; DQA6-MFI: 1,222) and the Flow-CDC result became negative (3.74%) (**Figure 3B**). At the same time, the second renal biopsy showed that both the glomerulitis and peritubular capillaritis were significantly improved (g0-1, ptc0-1) (**Figure 4B**). During the course of the treatment, the patient had no anorexia, fatigue, or other abnormal symptoms, as well as no abnormal changes in liver function or routine blood test results.

Since the treatment, the patient has been followed up for 1 year. At the tenth month of follow-up, the MFI values for DSA showed a rebound, especially in the anti-DQA6 antibody (DR12: 6,443, DQA6: 22,420). At the same time, the patient underwent a third renal biopsy. The pathology results showed that both glomerulitis and peritubular capillaritis remained mild (g1 and ptc1), and the C4d deposition was still negative (C4d0), but there was some progression in the chronic renal lesions (focal glomerular basement membrane double contours, mild interstitial fibrosis, and tubule atrophy; Banff 2017 scores: cg2, mm2, ci1, ct1; **Figure 4C**). The renal function remained quite stable during the whole follow-up period (**Figure 3D**). In the latest follow-up, his serum creatinine level was 133 μmol/L, and his total 24-hour urinary protein was 726 mg.

## Discussion

The development of *de novo* DSA after renal transplantation has been reported in 13%-30% of previously non-sensitized recipients (13). *De novo* DSAs are mainly directed to donor HLA class II mismatches and can appear at any time after kidney transplantation (14–16).The long-term existence of *de novo* DSA after renal transplantation has been reported to be associated with the occurrence of chronic AMR, transplant glomerulopathy, and late graft loss (17). The treatments targeting AMR vary widely, with most centers using a combination of PP and IVIG and a number of them also incorporating rituximab (18). However, there is no treatment currently proven to be effective in chronic AMR (4). In this study, we used splenic irradiation in addition to PP/IVIG and rituximab to treat chronic active AMR in two living-related kidney transplant recipients with class II *de novo* DSA production. After the treatment, the *de novo* DSA levels of both patients were significantly reduced, the acute pathological lesions of both allografts were partially reversed, and the progress of chronic lesions may have been delayed to a certain extent. Because patient 1 had an almost negative clinical response to the early treatment with rituximab in combination with 3 sessions of DFPP/IVIG and 3 sessions of PP/IVIG but showed a certain response on later treatment with repetitive low doses of splenic irradiation in addition to PP/IVIG, we think that the splenic irradiation may have played an important role in his improvement.

In the past, splenic irradiation was mainly used for treatment of chronic leukemias, myeloproliferative disorders, and some autoimmune diseases (19, 20). In the field of kidney transplantation, only one case report describes the application of splenic irradiation in the treatment of early severe acute AMR of two transplant recipients, which produced good clinical effects when used with a combination of PP/IVIG, rituximab, and eculizumab therapy (10).The immunological mechanisms by which splenic irradiation plays a role in the treatment of AMR are unclear. An animal study using a murine heart transplant model has demonstrated that the spleen is the major source of anti-donor antibody-secreting cells early after transplantation in both sensitized and non-sensitized recipients (21). Another interesting case report has described a high proportion of plasma cells in the resected spleen during the development of severe acute AMR (8). Thus, splenectomy or splenic irradiation can remove or deplete a large number of antibody-secreting cells in the spleen, which may partially explain why splenic irradiation is effective in the treatment of early acute AMR. However, for late *de novo* DSA-mediated chronic active AMR, the role of splenic irradiation apparently cannot be explained by the above-described mechanism. Unlike the sharp rise in DSA caused by an anamnestic response during acute AMR, *de novo* DSA leading to chronic AMR is usually maintained at a relatively stable level for a long time and is therefore considered to be mainly produced by the long-lived plasma cells in the bone marrow. Given that splenic irradiation itself has no direct effect on the niche plasma cells in the bone marrow, why it can reduce the level of DSA during the process of treating chronic active AMR is a novel immunological issue worthy of further investigation. We have hypothesized that the chronic activated B cells in the spleen and the long-lived plasma cells in the bone marrow jointly constitute a dynamic DSA production system to maintain a relatively constant DSA level for a long period of time. Therefore, repeated low-dose splenic irradiation over a relatively long period of time interfered with this DSA production system and thus played a certain role in reducing the DSA.

Although in the two cases we describe here, adjunct therapy with splenic irradiation resulted in a moderate decline in persistent class II DSA and a marked alleviation of acute pathologic lesions (e.g., glomerulitis and peritubular capillaritis), it is not possible to accurately evaluate whether the progression of chronic renal lesions (such as TG) was significantly prevented. In addition, we cannot isolate the effect of splenic irradiation in the setting of multiple therapies. Furthermore, a rebound in DSA levels occurred in both cases after 1 to 2 years of follow-up, suggesting that our treatment regimen did not achieve long-lasting effects, and further improvement of the regimen is needed.

To the best of our knowledge, this is the first case report detailing the use of splenic irradiation for the treatment of chronic active AMR in renal transplant recipients. This integrated treatment can potentially reduce DSA levels and alleviate both glomerulitis and peritubular capillaritis, which may help delay the progression of chronic AMR. Although two cases of effective treatment alone cannot define the effectiveness of a particular therapy, given the current inadequacy of conventional treatment, splenic irradiation may still be a promising option for the treatment of chronic active AMR.

## Data Availability Statement

The raw data supporting the conclusions of this article will be made available by the authors, without undue reservation.

## Ethics Statement

The studies involving human participants were reviewed and approved by the institutional review board at Tongji Hospital, Tongji Medical College, Huazhong University of Science and Technology. The patients/participants provided their written informed consent to participate in this study.

## Author Contributions

LZ took responsibility for the treatments of the patients, summarize clinical data, and drafting the manuscript. ZG participated in the treatment of the patients and collection of data. RS participated in data collection and drafting the manuscript. HG took responsibility for pathological diagnosis, histological images acquisition, and interpretation. JL made contributions in the treatment of plasmapheresis and double-filtration plasmapheresis. GC made substantial contributions to the study concept and design as well as revisions to the manuscript. All authors contributed to the article and approved the submitted version. 

## Funding

This work was supported by the Non-Profit Central Research Institute Fund of Chinese Academy of Medical Science (grant number 2019PT320014).

## Conflict of Interest

The authors declare that the research was conducted in the absence of any commercial or financial relationships that could be construed as a potential conflict of interest.

## Abbreviations

AMR, antibody-mediated rejection; CDC, complement-dependent cytotoxicity; DSA, donor-specific antibody; DFPP, double filtration plasmapheresis; IVIG, intravenous immunoglobulins; MFI, mean fluorescence intensity; PP, plasmapheresis; PRA, panel reactive antibodies; TG, transplant glomerulopathy
